# Outcomes and predictors of failure of arteriovenous fistulae for hemodialysis

**DOI:** 10.1007/s11255-021-02908-5

**Published:** 2021-06-06

**Authors:** Siddharth Venkat Ramanan, Ravindra Attur Prabhu, Indu Ramachandra Rao, Arun Chawla, Srinivas Vinayak Shenoy, Shankar Prasad Nagaraju, Mohan V. Bhojaraja

**Affiliations:** 1 Department of Nephrology, Kasturba Medical College Manipal, Manipal Academy of Higher Education, Madhav Nagar, Manipal, 576104 India; 2 Department of Urology, Kasturba Medical College Manipal, Manipal Academy of Higher Education, Madhav Nagar, Manipal, 576104 India

**Keywords:** Arteriovenous fistula, Vascular access, Failure, Hemodialysis

## Abstract

**Purpose:**

Arteriovenous fistula(AVF) is preferred vascular access for hemodialysis but has primary failure in 20–60%. Studying predictors of AVF failure would help plan appropriate management.We studied AVF outcomes, clinical and vascular factors predicting their failure in patients requiring hemodialysis.

**Methods:**

Retrospective study of patients with AVF creation from January 2017 to December 2018. Outcomes studied were immediate (< 72 h), primary (3 months) AVF failure, six-month/one-year patency, analyzed for predictive clinical, vascular factors as assessed using Pre-operative Doppler Ultrasound(DUS).

**Results:**

Of 530 AVFs in 460 patients, DUS was done in 426/530 (80.4%), 349/460 (75.8%) were males, mean age was 53.10 ± 14.54 (18–91), 215/460(46.7%) had Diabetes mellitus(DM), 423/460(92%) hypertension. AVFs were radiocephalic in 79/530 (14.9%), brachiocephalic 418/530 (78.9%), brachiobasilic 33/530 (6.2%). AVF Immediate/Primary failure was seen in 64/530 (12.1%), 90/352 (25.6%); Patency at six months/one year in 253/352(71.8%),191/305 (62.6%), respectively. Older age had less immediate failures (AOR 0.97, CI 0.95–0.99, *p* 0.03). Feeding arterial diameter predicted immediate and primary failure on univariate analysis [OR 0.64 (95% CI 0.49–0.83), 0.62 (95% CI 0.47–0.89), respectively], but not multivariate. Artery diameter of > 4.0 mm had less failures [immediate (*p* 0.01), primary (*p* 0.02)], < 2.0 mm had specificity 95.9% and 95.4% for immediate, primary failure respectively.

**Conclusion:**

AVF failure is 12.1%, immediately; 25.6% three months after construction, Patency at 6 months is 71.8%, one year 62.6%. Immediate failures decrease with age. Artery diameters > 4.0 mm had less, < 2.0 mm had more failures.

## Introduction

Arteriovenous fistula (AVF) of native vessels as vascular access for hemodialysis is preferred over prosthetic grafts and central venous catheters because, at comparable flow rates, it is associated with lower mortality, infections and cardiovascular events [1]. Maturation of AVF depends on adequacy of vessels and time allowed before use. Since this is a lifeline for patients, pre-operative evaluation is important. Doppler ultrasonography (DUS) is standard for vascular evaluation as it evaluates both structure and function of peripheral vessels and, hence, is useful for pre-operative planning of AVF [2]. However, guidelines do not recommend its use in all patients prior to AVF construction and definite vascular parameters have not been established [3]. We studied early outcomes of AVFs in our center and clinical and vascular factors predicting their failure.

## Materials and methods

### Study design: retrospective cohort study

Patient population and clinical resources: The study was approved by the institutional ethics committee. Case records of consecutive patients who had AVF construction at our center between Jan 2017 and December 2018 were perused for demographic details, type of AVF and pre-operative DUS measurements. Patients younger than 18 years were excluded. Comorbidities noted were Ischemic heart disease(IHD), diabetes mellitus(DM), hypertension, cerebrovascular accidents(CVA), and peripheral arterial disease(PVD) using standard definitions. Vascular characteristics noted were anteroposterior (AP) diameter of brachial, ulnar and radial arteries; venous AP diameter, compressibility in basilic and cephalic veins. Physical examination was carried out by the operating urologist, pulses at elbows and wrists, and the superficial veins in the forearm and upper arm were assessed.

The DUS examination was conducted by a radiologist using a Phillips Affiniti 70 Ultrasound system with a 6–13 Megahertz linear probe with angle of insonation less than 60 degrees. Coronal views were used for measurements of diameter of all blood vessels and longitudinal views for measuring blood flow velocities with patients sitting upright with arm extended below heart level.

#### Vein assessment

Gray-scale Doppler ultrasound was used to assess for obvious scarring from previous venepuncture, for compressibility of the vein from the wrist to the axilla, and for measurement of vein diameter. The cephalic and basilic veins were routinely mapped.

#### Arterial measurements

Gray-scale Doppler ultrasound is used to assess for obvious vascular scarring or calcification. Moderate vascular calcification did not preclude AVF creation, although severe radial artery calcification was regarded as a contraindication to RAVF. The radial or brachial arterial diameters were measured at wrist and antecubital fossa, respectively, during systole. Peak arterial velocity was measured in both radial and brachial arteries using color Doppler ultrasound in the longitudinal plane, approximately 5 cm from the wrist crease and antecubital fossa, respectively, to allow a good straight segment of blood vessel for analysis.

AVFs were created by a single urologist trained in the procedure. Patient follow-up was achieved in outpatient or through telephonic contact of their health care givers.

Outcomes: Primary outcomes were immediate failure defined as an AVF that has either no appearance of or loss of bruit or thrill within 72 h of creation and primary failure defined as an AVF that was inadequate for hemodialysis at 3 months’ post creation, including immediate failure, failure to mature or early thrombosis [4]. Secondary outcomes were cumulative survival at 6 and 12 months defined as the time of access creation or placement until access abandonment or achievement of a censored event (death, transfer to peritoneal dialysis, transplantation, and end of study period), and included all surgical and endovascular interventions [5]. Hypothesis tested was effect of clinical factors and target arterial and venous diameters as measured by DUS on immediate and primary failure of AVF.

Statistical analysis: Statistical analysis was performed using SPSS version 23 (IBM Corp., Armonk, NY, USA). Descriptive data were presented as frequency (*n*) and percentages (%) for categorical data, and mean ± standard deviation (SD) for continuous variables. Comparison of proportions was done by chi-square test and continuous data by t-test. To identify factors predictive of immediate and primary AVF failure, univariate and multivariate logistic regression analyses were done. Crude and adjusted odds ratios were calculated, with 95% Confidence Intervals (CI). Receiver operating characteristic curves were generated to determine the predictive value of pre-operative target artery and vein diameters for immediate and primary failures. P values of < 0.05 were considered as statistically significant.

## Results

**Fig. 1:**
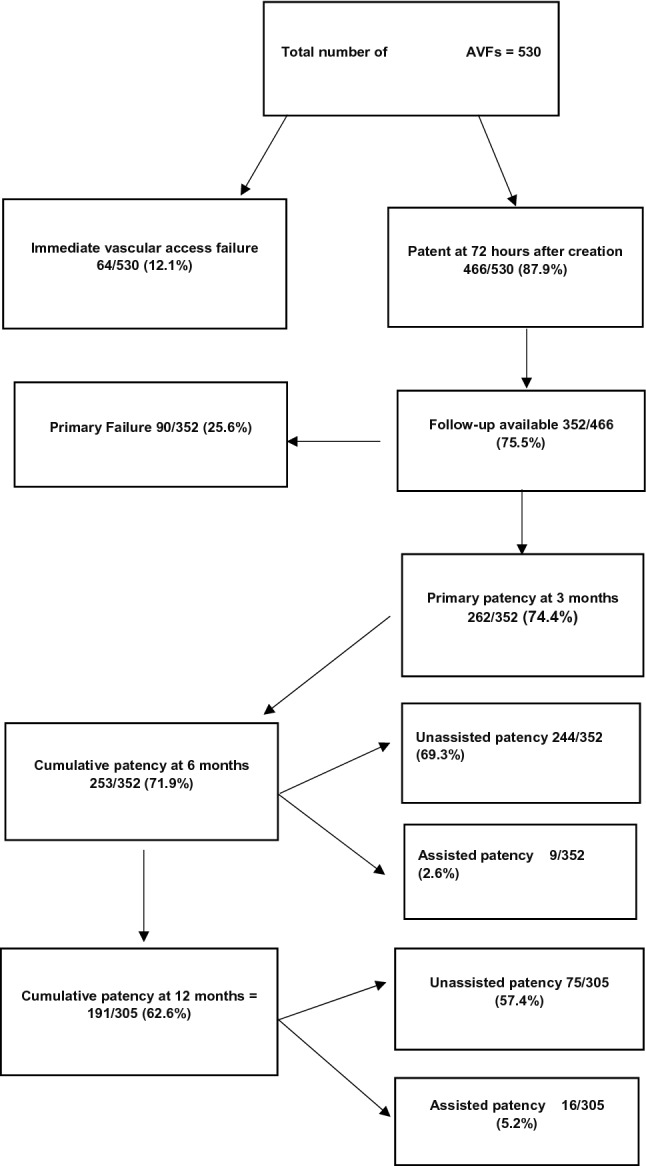
Outcomes of arteriovenous fistulae

**Fig. 2:**
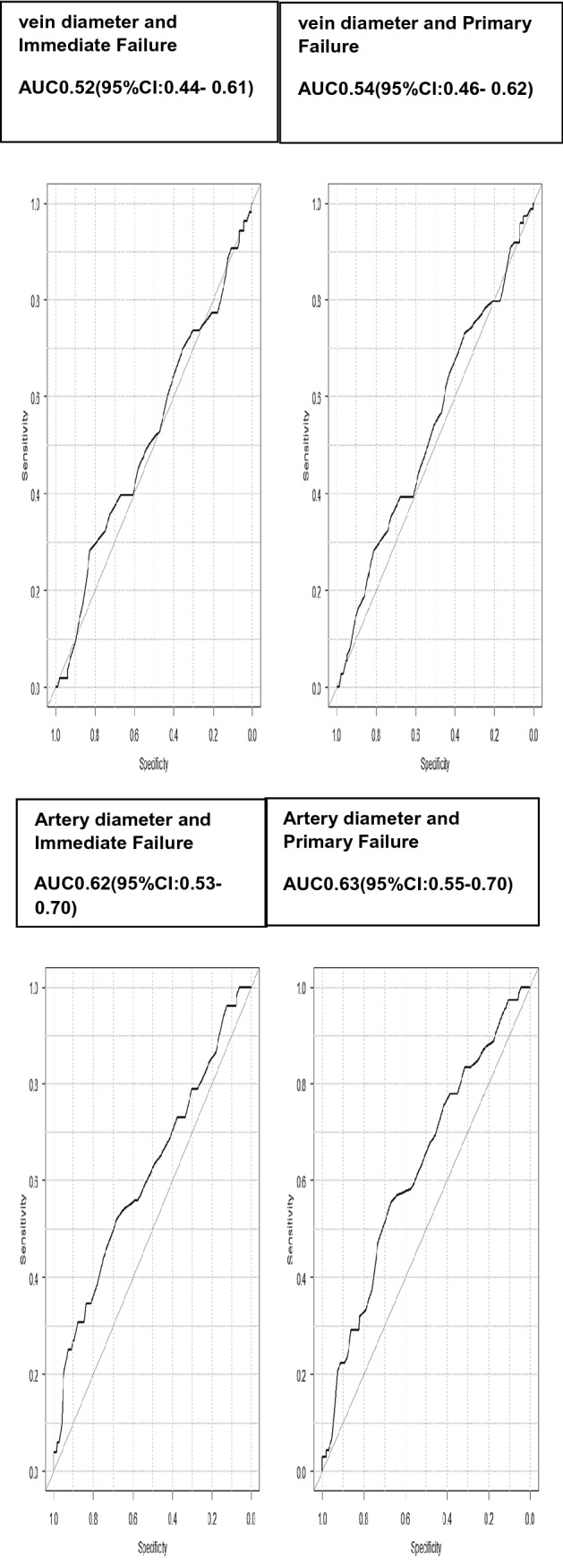
ROC analysis

Of 530 AVFs created in 460 patients, pre-operative duplex ultrasound was available for 426/530(80.4%) in 377/460 (82%) patients. Baseline characteristics are in Table 1. Of 460 patients, 349 (75.8%) were males, mean age was 53.10 ± 14.54 (18–91), 215(46.7%) had diabetes mellitus, 423(92%) had hypertension, 55(12%) had ischemic heart disease, 16(3.5%) had cerebrovascular accident and nine (2%) had peripheral vascular disease. Types of AVF done were radiocephalic in 79/530 (14.9%) [left 73 (13.8%)], brachiocephalic in 418/530 (78.9%) [left 345 (65.1%)], brachiobasilic in 33/530 (6.2%) [left 18 (3.4%)]. At three months, outcome data was available for 352 AVFs of which 292(83%) had pre-operative ultrasound Doppler. Immediate failure was seen in 64/530 (12.1%) and primary failure 90/352 (25.6%). At six months and one year, AVF patency was seen in 253/352(71.8%), unassisted in 244/352(69.3%) and 191/305 (62.6%), unassisted in 175/305(57.4%), respectively (Fig. 1 Outcomes of Arteriovenous Fistulae). Association of DUS with patient characteristics for radiocephalic and upper arm AVFs is given in Table 2, and Predictive factors for AVF outcomes in Table 3. Overall males (*p* = 0.04) had higher arterial diameters. In upper arm males (p < 0.005), age more than 65 years (*p* = 0.005), those with IHD (*p* = 0.04) had higher arterial diameters and DM was associated with both higher arterial and venous diameters (both *p* < 0.001). Increasing age was associated with less immediate failures(AOR 0.97,CI 0.95–0.99, p 0.03).Feeding artery diameter of > 4.0 mm was significantly associated with less immediate (p 0.01) and primary failures (*p* 0.02). On receiver operating curve analysis(Fig. 2 ROC Analysis for Immediate, Primary Failure and Pre-operative Artery and Vein Diameter), arterial diameters did not predict immediate and primary failure with an Area Under Curve(AUC) of 0.615 (95% CI 0.528–0.70) and 0.625 (95% CI 0.55–0.70), respectively. Arterial diameter of < 2.0 mm had sensitivity of 9.6% and 6.9% for immediate and primary failure, respectively, but had a specificity of 95.9% for immediate and 95.4% for primary failure. Pre-operative venous diameter did not predict immediate and primary failure with an AUC 0.524 (95% CI 0.437–0.61) and 0.539 (95% CI 0.462–0.617), respectively. Venous diameter of > 2.5 mm had a sensitivity and specificity of 45.3% and 57.9% for immediate failure, and 44.6% and 58.3% % for primary failure.

**Table 1:** Baseline characteristics of patients and arteriovenous fistula

Patient characteristics	Number = 460(%)
Age, years^*^	53.10 ± 14.54
Male gender	349 (75.8)
Diabetes mellitus	215(46.7)
Hypertension	422 (91.7)
Ischemic heart disease	55(12)
Cerebrovascular disease	16(3.5)
Peripheral vascular disease	9(2)

Arteriovenous Fistula characteristics	Number = 530
Upper arm fistula	451 (85.1)
Brachiocephalic	418 (78.9)
Left	348 (65.7)
Right	70 (13.2)
Brachiobasilic	33 (6.23)
Left	18 (3.4)
Right	15 (2.8)
Radiocephalic	79(14.9)
Left	73 (13.8)
Right	6 (1.1)
Pre-operative vascular ultrasound)	426 (80.4)

^*^mean ± SD

**Table 2:** Association of patient characteristics with pre-operative Doppler ultrasound

Patient characteristics		Mean arterial diameter (mm)	*P* value		Mean venous diameter (mm)	*P* value
Radiocephalic arteriovenous fistulae						
Age, years						
< 65		2.42 ± 0.69	0.84		3.14 ± 1.23	0.07
≥ 65		2.35 ± 0.79			2.00 ± 0.84	
Gender						
Male		2.45 ± 0.68	**0.04**		3.12 ± 1.22	0.06
Female		1.45 ± 0.21			1.45 ± 0.63	
Diabetes Mellitus						
Yes		2.44 ± 0.80	0.83		2.38 ± 1.17	0.17
No		2.40 ± 0.61			2.00 ± 0.97	
Hypertension						
Yes		2.44 ± 0.70	0.32		3.10 ± 1.24	0.27
No		2.03 ± 0.06			2.30 ± 1.21	
Ischemic heart disease						
Yes		3.08 ± 1.15	0.05		2.95 ± 0.70	0.85
No		2.37 ± 0.64			3.08 ± 1.27	
Upper arm arteriovenous fistula						
Age, years						
< 65	4.19 ± 0.85		**0.005**	2.85 ± 1.10		0.12
≥ 65	4.50 ± 0.89			3.05 ± 1.05		
Gender						
Male	4.42 ± 0.87		** < 0.001**	2.89 ± 1.10		0.67
Female	3.86 ± 0.70			2.83 ± 1.01		
Diabetes Mellitus						
Yes	4.41 ± 0.81		**<0.001**	3.10 ± 1.14		** < 0.001**
No	4.10 ± 0.89			2.69 ± 1.02		
Hypertension						
Yes	4.28 ± 0.86		0.38	2.90 ± 1.11		0.36
No	4.08 ± 0.91			2.67 ± 0.94		
Ischemic Heart disease						
Yes	4.52 ± 0.71		**0.04**	2.94 ± 0.96		0.77
No	4.24 ± 0.88			2.89 ± 1.12		
Cerebrovascular accident(CVA)^a^						
Yes	4.43 ± 0.86		0.51	2.89 ± 1.01		0.50
No	4.27 ± 0.87			3.10 ± 1.26		
Peripheral vascular disease(PVA)^a^						
Yes	4.62 ± 0.66		0.22	2.93 ± 0.89		0.89
No	4.26 ± 0.87			2.87 ± 1.08		

a Patients with CVA/PVD all underwent upper arm AVFs

**Table 3:** Arteriovenous fistula outcome predictors

	Number/total (%)				
Feeding artery diameter	< 2 mm	2–2.9 mm	3–3.9 mm	> 4 mm	*P* value
Immediate failure^*^	3/13(23.1)	13/50(26)	13/123(10.6)	23/237(9.7)	**0.01**
Primary failure^*^	3/9(33.3)	18/43(41.8)	22/87(25.3)	29/150(19.3)	**0.02**

Feeding vein diameter	< 2.5 mm	2.5–3.4 mm	3.5–4.4 mm	> 4.5 mm	*P* value
Immediate failure	21/167(12.6)	18/132(13.6)	9/89(10.1)	5/38(13.2)	0.90
Primary failure	29/113(25.6)	27/97 (27.8)	12/60(20)	6/22(27.3)	0.73

Covariate	Unadjusted OR (95% CI)	*P* value	Adjusted OR (95% CI)	*P* value
Immediate failure(number 426)				
Age	0.97 (0.95–0.99)	**0.003**	0.97 (0.95–0.99)	**0.03**
Male gender	0.74 (0.41–1.32)	0.30	–	–
Diabetes	0.74 (0.43–1.25)	0.26	–	–
Hypertension	1.08 (0.31–3.70)	0.90	–	–
IHD	0.24 (0.06–1.02)	0.05	0.45 (0.10–2.03)	0.30
CVA	0.45 (0.06–3.43)	0.44	–	–
PVD	5.11 (1.40–18.60)	**0.01**	10.0 (2.44–41.30)	**0.001**
Proximal AVF	0.35 (0.19–0.64)	** < 0.001**	0.44 (0.16–1.15)	0.09
Left-sided AVF	0.77 (0.36–1.61)	0.48	–	–
Artery diameter (mm)	0.62 (0.47–0.89)	**0.001**	0.84 (0.56–1.3)	0.41
Vein diameter (mm)	0.96 (0.74–1.25)	0.75	–	–
Primary failure(number 292)				
Age	0.98 (0.92–0.99)	**0.01**	0.98 (0.96–1.00)	0.12
Male gender	0.61(0.35–1.04)	0.07	0.88 (0.44–1.76)	0.72
Diabetes	0.96 (0.59–1.60)	0.88	–	–
Hypertension	1.73 (0.49–6.11)	0.39	–	–
IHD	0.36 (0.13–1.06)	0.07	0.654 (0.21–2.01)	0.45
CVA	0.73 (0.08–6.62)	0.78	–	–
PVD	2.39 (0.63–9.11)	0.20	–	**–**
Proximal AVF	0.48 (0.27–0.86)	**0.02**	0.91 (0.34–2.40)	0.84
Left-sided AVF	0.84 (0.46–1.54)	0.57	–	–
Artery diameter (mm)	0.64 (0.49–0.83)	**0.001**	0.72 (0.48–1.06)	0.09
Vein diameter (mm)	0.89 (0.70–1.14)	0.36	–	–

^*^Data on artery diameter not available for three

## Discussion

Deliberate connection between an upper extremity artery and vein, i.e., AVF is the preferred vascular access for maintenance hemodialysis in patients with CKD stage 5D. AVFs have superior patency and lower complications compared with other vascular access types. In routine practice, vessel suitability for fistula placement is determined by clinical examination.

A retrospective study[5] of 599 patients with 289 AVFs and 310 arteriovenous grafts(AVG) identified female gender to be associated with higher requirement of interventions in AVFs before successful cannulation and requirement of prior intervention and older age to be the factors predicting failure after a successful cannulation.

Contrary to expectation, younger age was associated with more immediate failures of AVF in our study and presence of DM did not influence overall outcomes. This may be representative of our population but further prospective studies with stratification for age are warranted. Similar to above-mentioned study, gender and presence of Ischemic heart disease, cerebrovascular accident did not influence outcomes probably indicating overall influence of vessel size. Since vessel size and patency may to some extent be assessed by physical examination role, Doppler ultrasound may be superfluous except in special circumstances. We found patency rates of 87.9%, 74.4%, 71.8%, 62.6% immediately, at three and six months and one year, respectively, with 2.5% and 5.4% requiring assistance to maintain patency at six months and one year, respectively. This is somewhat similar to the results presented in a systematic review[6] including 380 studies which reported primary unassisted, primary assisted and secondary patency rates of 64%,73%,79%, respectively, with 26% AVFs maturing at six months and 21% abandoned before use.

Studies comparing use of pre-operative Doppler ultrasound with clinical exam have found no differences in primary AVF patency, primary failure, requirement of postoperative intervention [7, 8] but a higher secondary patency rates[9].

Hence, KDOQI clinical practice guideline for vascular access recommends use of pre-operative DUS in patients at high risk of AVF failure such as advanced age, obesity, female gender, peripheral vascular disease, and coronary artery disease; rather than routine vascular mapping [3]. This is based on a small randomized control study comparing selective and routine vessel mapping which did not show a difference in AVF failure at 90 days [10]. These results are similar to our findings which show extremes of arterial diameter are predictive of patency. Studies assessing utility of Doppler assessment are sparse and parameters not defined. Multicenter dialysis access consortium fistula study reported a primary failure of 60% in spite of routine use of Doppler ultrasound pre-operatively [11]. A meta-analysis of five randomized controlled trials with 574 patients showed lower immediate AVF failure rates in patients who underwent upper extremity Doppler ultrasonographic examination prior to surgery but this did not offer a significant advantage over clinical examination [12]. One study recommended routine use of Doppler based on comparison with a retrospective cohort showing reduced primary failure due to a multidisciplinary experienced team performing the AVF [13]. Primary AVF failure rate in our study was 25.6%; it typically ranges from 23 to 40% [14, 15].Wilmink et al. noted that vessel diameter did not predict AVF functionality and should not be used to avoid construction [16]. Arterial diameter of > 4.0 mm was associated with better outcomes in our study. In a study by Farrington et al., arterial diameter was observed to be an important predictor for both unassisted and overall AVF maturation [17]. Farber et al. demonstrated that early thrombosis risk was higher in women than in men, for forearm than upper arm fistulas, for radial than brachial artery fistulas, and for fistulas constructed from smaller caliber arteries or veins, although the trend for veins surprisingly did not extend below vessels 3 mm in diameter [18].

In our study, there was a trend for feeding artery diameter in predicting immediate and primary failure, those with arterial diameters > 4.0 mm had lesser and < 2.0 mm had more failures, respectively. Majority of our diabetic patients have undergone upper arm AVFs, which is likely why they appear to have higher diameters and blood flow. Similarly, 95.57% of those aged 65 and above, and 94.7% of IHD patients underwent upper arm AVFs.

Strengths of our study are the large number of AVFs done at a single center. Limitations are retrospective nature, loss to follow-up and Doppler studies not being done by the surgeon constructing the AVFs.

## Conclusion

AVF failure is seen in 12.1% immediately after construction and in 25.6% at three months. AVF patency rates are 71.8% at 6 months and 62.6% at one year. Artery diameters of > 4.0 mm had less and < 2.0 mm had more failures. Younger age is associated with higher immediate failures.

## Data Availability

The datasets generated during and/or analyzed during the current study are available from the corresponding author on reasonable request.
